# From Buddhist meditation to psychological well-being: an ethical-cultural training ecology model

**DOI:** 10.3389/fpsyg.2026.1938434

**Published:** 2026-09-03

**Authors:** Yukun Mei, Jiyu Li, Sarawadee Phuchomsri

**Affiliations:** 1 School of Music and Dance, Xihua University, Chengdu, Sichuan, China; 2 Faculty of Fine and Applied Arts, Khon Kaen University, Khon Kaen, Thailand

**Keywords:** Buddhist meditation, Buddhist-contextualized mindfulness, compassion, emotion regulation, nonattachment, psychological well-being, self-decentering, training ecology

## Abstract

Buddhist meditation is widely studied in relation to mindfulness, emotion regulation, compassion, and well-being, yet it is often operationalized as an attentional or stress-reduction technique detached from the ethical and cultural conditions of practice. This article develops the ethical-cultural training ecology model, a Hypothesis and Theory framework that treats Buddhist meditation as a coordinated practice ecology rather than a single psychological technique. The model distinguishes four nested analytic levels. Ethical-cultural ecology provides the process-shaping context; a probabilistic functional hierarchy links attention, emotion regulation, self-decentering, nonattachment, and compassionate reorientation; authority reorganization provides a higher-order explanatory principle; and multidimensional well-being defines the distal outcome domain. Its distinctive claim is relational rather than additive: familiar mechanisms are hypothesized to acquire different functions through asymmetric dependencies and ecological configuration. Attention may increase observability, emotion regulation may increase affective tolerability, self-decentering may demote self-related narratives from self-truth, nonattachment may weaken their action-guiding grip, and compassion may provide ethical-relational direction while recursively supporting emotional safety. Authority reorganization is not proposed as an independent latent mechanism. Self-transcendence is treated as a possible experiential and relational outcome. Three theory-driven hypothetical illustrations specify predicted and disconfirming patterns, and five propositions translate the framework into prospective, incremental, mediation, and configuration-level hypotheses for future empirical testing.

## 1 Introduction

Modern psychology has engaged with Buddhist meditation largely through mindfulness-based clinical programs, acceptance- and compassion-oriented approaches, contemplative neuroscience, and positive psychology. Kabat-Zinn’s work made mindfulness clinically visible as a disciplined form of awareness (Kabat-Zinn, 1990, 2003), while mindfulness-based cognitive therapy extended this translation into relapse prevention for depression (Segal et al., 2002). Later reviews and neuroscience-oriented accounts further strengthened the behavioral, psychometric, and neural study of meditation (Baer, 2003; Hölzel et al., 2011; Tang et al., 2015). Yet this successful translation has also narrowed the object under study. Meditation is often described chiefly as present-moment awareness, attention regulation, or nonjudgmental observation. These are indispensable elements, but they leave out much of the Buddhist path: ethical orientation, insight, nonattachment, compassion, and liberation from habitual reactivity.

This narrowing is not merely definitional; it may affect what kinds of psychological change research is prepared to detect. Concentration may improve while self-criticism remains intact, distress may be noticed while the person remains fused with it, and mindfulness may be used instrumentally for productivity rather than as a reorientation toward suffering (Bodhi, 2011; Van Dam et al., 2018; Berger et al., 2024). The concern is not that secular mindfulness is invalid, but that bracketing ethical, cultural, and wisdom-oriented frames may alter the psychological function of practice. The central question is therefore whether familiar mechanisms retain the same function when removed from, translated from, or re-embedded within Buddhist ethical-cultural training.

The present article develops the ethical-cultural training ecology model of Buddhist meditation and psychological well-being. It treats meditation as a coordinated configuration in which ethical orientation, attentional stabilization, affective regulation, insight into self-processes, nonattachment, and compassion can mutually shape one another. The model is a psychological abstraction from selected Buddhist-derived practices, not a universal account of Buddhist traditions. Its unit of analysis is a coordinated practice configuration rather than a tradition considered as a whole. Three practice-anchored theoretical analytic illustrations – mindfulness of breathing and anxious self-narrative, loving-kindness practice and interpersonal resentment, and Buddhist-contextualized mindfulness as a threefold-training ecology – make the model’s logic available for critical examination and future hermeneutic, interview-based, narrative, clinical, and practice-process research. They are constructed interpretive scenarios, not empirical reports or secondary analyses of other authors’ data.

Terminological clarification is necessary. In this article, Buddhist meditation refers to the broader practice ecology that includes ethical orientation, attentional cultivation, insight, nonattachment, loving-kindness, and compassion. Mindfulness is used more narrowly to refer either to present-centered awareness within Buddhist practice or to the psychological construct used in modern mindfulness research. Secular mindfulness refers to clinical or educational programs, such as MBSR and MBCT, when they bracket, translate, or operationalize Buddhist doctrinal and ethical contexts for therapeutic or educational purposes. Buddhist-contextualized mindfulness does not refer to Asian or Buddhist settings in general. It refers here to mindfulness situated within a threefold-training ecology of morality (sila), concentration (samadhi), and wisdom (panna), together with teacher or community guidance, repeated practice, and ethical feedback when these are present. These distinctions are maintained to avoid treating mindfulness and Buddhist meditation as interchangeable terms.

Within this model, training ecology names the process-shaping context; functional hierarchy names the probabilistic organization of mechanisms; authority reorganization names the unifying explanation of self-related change; and multidimensional well-being names the distal outcome domain. These are nested analytic levels rather than competing models. The central movements are attentional stabilization, affective regulation, self-decentering and nonattachment, and compassionate reorientation; self-transcendence is treated as a possible experiential and relational outcome.

Drawing on Buddhist-Western psychological dialogue, contemporary contemplative science, and selected Chinese-language interpretive resources, the article makes five contributions. First, it specifies probabilistic and asymmetrical dependencies among attentional observability, affective tolerability, self-related transformation, and compassion. Second, it operationalizes training ecology through five provisional dimensions that capture normative, interpretive, social, temporal-institutional, and corrective conditions of practice. Third, it distinguishes metacognitive self-decentering from affective-motivational nonattachment. Fourth, it treats changes in the truth-status and action-guiding force of experience as a higher-order explanatory problem of authority reorganization. Fifth, it derives falsifiable propositions and boundary conditions from practice-anchored illustrations without presenting constructed scenarios as empirical evidence.

Several influential models provide essential comparative reference points. Hölzel et al. (2011) decomposed mindfulness into attention regulation, body awareness, emotion regulation, and changes in perspective on the self. Shapiro et al. (2006) emphasized intention, attention, attitude, and reperceiving. Vago and Silbersweig (2012) S-ART framework organized mindfulness around self-awareness, self-regulation, and self-transcendence. Mindfulness-to-meaning theory linked mindfulness with positive reappraisal, savoring, and upward spirals of well-being (Garland et al., 2015, 2022; Garland and Fredrickson, 2019). The present framework does not claim priority for these component mechanisms. Its novelty is evaluated along two narrower axes: whether mechanisms are assigned asymmetrical functional dependencies rather than merely co-listed, and whether ethical-cultural ecology is treated as a formative condition that can alter the psychological function of otherwise similar practices. This positioning makes the model testable against established accounts rather than simply more inclusive than them.

Table 1 summarizes the comparison on these two axes. The model’s distinctive prediction is not merely that attention, emotion regulation, self-decentering, nonattachment, compassion, and well-being covary. It predicts that comparable attentional gains can have different downstream consequences depending on whether affect becomes tolerable, self-related content loses epistemic or motivational authority, and practice is embedded in ethical, interpretive, social, repetitive, and corrective supports. This distinction is intended to explain patterns such as improved attentional control with persistent self-fusion, short-term stress relief without durable self-related change, or technically competent mindfulness used primarily for performance optimization rather than relational or ethical transformation.

**TABLE 1:** Comparative positioning of the present framework along relational and ecological axes.

Model or framework	Core architecture	Comparative axes: hierarchy / ecology	Distinctive contrast / prediction
Hölzel et al., 2011	Attention; body awareness; emotion regulation; self-perspective.	Hierarchy: decomposed, not asymmetric. Ecology: not central.	Enabling vs. transformative functions; ecology-dependent divergence after similar attentional gains.
Shapiro et al., 2006	Intention; attention; attitude; reperceiving.	Hierarchy: relational; no decentering/nonattachment split. Ecology: limited.	Separates decentering from nonattachment; maps them to epistemic vs. motivational authority.
Vago and Silbersweig, 2012	Self-awareness; self-regulation; self-transcendence (S-ART).	Hierarchy: integrative. Ecology: not a primary analytic level.	Self-transcendence as outcome; adds authority reorganization and ecology-dependent mechanism function.
Garland et al., 2015, 2022; Garland and Fredrickson, 2019	Mindfulness-to-meaning; reappraisal; savoring; upward spirals.	Hierarchy: directional. Ecology: meaning central; corrective context not primary.	Tests incremental nonattachment/ecology; predicts divergence in relational durability beyond meaning-making.

The framework therefore specifies competing explanations. An additive account would be favored if the proposed mechanisms contributed independently and retained the same functions across ecological configurations. A mindfulness-to-meaning account would be sufficient if the same self-related and relational outcomes were explained without incremental contributions from nonattachment, authority reorganization, or ecological configuration. A clinical-skills account would be sufficient if durable change were fully explained by attentional and regulatory skill acquisition without measurable changes in the truth-status or action-guiding force of self-related narratives. These alternatives define comparative tests through which the present framework can be supported, delimited, or revised.

Across these comparisons, the defining question of the present framework is how familiar mechanisms change their psychological function when organized within an ethical-cultural training ecology.

## 2 Buddhist meditation as a psychological training ecology

### 2.1 Meditation beyond technique

In contemporary psychological research, mindfulness is often defined as present-centered, nonjudgmental awareness (Kabat-Zinn, 2003) or as a two-component process involving attention regulation and an attitude of openness, curiosity, and acceptance (Brown and Ryan, 2003; Baer et al., 2006; Bishop et al., 2004). These definitions are useful for measurement, but they are narrower than Buddhist meditation as practiced and theorized across Buddhist traditions. Buddhist meditation includes attentional stabilization, ethical preparation, insight into impermanence and non-self, nonattachment from craving and aversion, loving-kindness, compassion, and wisdom (Analayo, 2003; Bodhi, 2011; Gethin, 2011). The model should therefore be read as a theoretical abstraction rather than a doctrinal map of Buddhism as a whole. Its scope is psychological and interpretive: it identifies a provisional configuration of functional relations across selected Buddhist concepts and practice forms, not a universal account of every Buddhist school, ritual setting, or meditative technique.

The term training ecology is used here because meditation is not a single mental operation. It is a system of practices, values, interpretations, and experiential skills that reinforce one another. In Buddhist terms, attention training is not value-neutral; it is cultivated within a normative context that seeks to reduce greed, hatred, and delusion. This conceptual definition is intentionally broad: it prevents meditation from being reduced either to religious doctrine alone or to a technique of attention alone, while the observable dimensions of the ecology are specified later in Section “3.3 Operationalizing the training ecology”.

This broader reading also draws on Buddhist-Western psychological dialogue and contemporary contemplative science. Wallace and Shapiro (2006) frame mental balance and well-being as a bridge between Buddhist psychology and Western psychological inquiry. Dahl et al. (2020) similarly describe well-being as trainable through interrelated dimensions of awareness, connection, insight, and purpose. This international literature provides the broader comparative frame within which Xiong G.’s account of Buddhist philosophy of mind is used as a culturally specific conceptual enrichment rather than as the sole theoretical foundation. Xiong (2014) distinguishes between inquiry into the structure of mental phenomena and inquiry into the use, cultivation, and resources of mind, including happiness, suffering, emotional life, freedom, and liberation. For the present article, this distinction is useful because it prevents a purely descriptive reading of Buddhist mind theory. Buddhist psychology is concerned not only with what mental events are, but also with how they entangle a person in suffering or become part of a path toward release.

### 2.2 Selected Chinese-language interpretive resources

The international literature reviewed above provides the primary theoretical foundation. Selected Chinese-language discussions are used more narrowly as culturally situated interpretive resources for assumptions about cognition, selfhood, suffering, and change. Xiong (2011) highlights what may be lost from accounts of etiology, healing, cognition, and self when mindfulness is detached from an underlying view of the person. Xiong (2014) broadens the frame toward the cultivation and use of mind. Xiao (2018) provides a vocabulary of self-insight, nonattachment, self-compassion, and self-acceptance. These sources are not treated as representative of East Asian Buddhism, as pan-Buddhist authorities, or as independent evidence for the model.

Their role is interpretive rather than evidential. Psychologically, a less divided or less attached mode of being may be translated cautiously as reduced cognitive fusion, experiential avoidance, rigid self-identification, and chronic reactivity, together with greater decentering, nonattachment, self-compassion, and flexibility. This translation connects culturally situated assumptions about selfhood and suffering to observable psychological and qualitative indicators, while preserving the distinction between Buddhist concepts and their psychological proxies.

### 2.3 Translating Buddhist concepts for psychological inquiry

Table 2 presents the provisional conceptual correspondences between selected Buddhist concepts and psychological inquiry.

**TABLE 2:** Provisional conceptual correspondences between selected Buddhist concepts and psychological inquiry.

Buddhist concept	Provisional psychological analogue	Qualitative interpretive indicators
Sati / mindfulness	Present-centered awareness, monitoring of mental states, and repeated return from distraction	Narratives of noticing wandering, returning to breath/body, and distinguishing awareness from thought-content
Samadhi / concentration	Stabilized attention and continuity of awareness	Descriptions of steadiness, reduced scattering, greater capacity to remain with one object or field of experience
Vipassana / insight	Seeing thoughts, emotions, and self-narratives as conditioned and transient processes	Language of arising, passing, contingency, impermanence, and seeing a thought as a thought rather than as truth
Nonattachment	Loosening of clinging, craving, avoidance, and rigid self-identification	Reduced compulsion to defend a self-story; increased ability to let experience be present without grasping or rejection
Anatta / non-self	Reduced reification of the self; the self is experienced as conditioned, processual, and non-absolute.	Shift from “this is what I am” to “this experience is arising under conditions”; less reification of roles and emotions
Metta / karuna	Loving-kindness, compassion, and relational concern	Movement from self-protection or resentment toward care, bounded goodwill, and recognition of shared vulnerability
Sila / ethical orientation	Non-harm, responsibility, and value-based framing of practice	Practice linked to speech, action, boundaries, and concern for others rather than private self-optimization alone

**TABLE 3:** Operational dimensions of the Buddhist ethical-cultural training ecology.

Ecological dimension	Operational definition	Process-shaping function	Possible qualitative or comparative material
Ethical directedness	Practice is explicitly oriented toward non-harm, responsibility, compassion, and reduced reactivity.	May direct attention and regulation toward suffering and care rather than private self-optimization alone.	Session transcripts; audio or video recordings; teacher-training manuals; precept instructions; and practitioner accounts of practice purpose and non-harm.
Meaning framework	Practice is embedded in interpretations of suffering, impermanence, non-self, interdependence, liberation, or flourishing.	May change how difficult emotions and self-narratives are interpreted and worked with.	Meditation manuals; session curricula; doctrinal or explanatory texts; interview and diary narratives; and clinical framing documents concerning suffering and change.
Community mediation	Practice is supported by teachers, peers, rituals, shared vocabulary, and collective participation.	May provide social scaffolding, guidance, normalization, and accountability for difficult practice processes.	Group observations; attendance records; peer-support interactions; teacher-student exchanges; ritual or community participation records; and retreat fieldnotes.
Institutionalized repetition	Practice has stable temporal, spatial, and procedural forms, such as regular sitting, chanting, retreats, or daily routines.	May help translate occasional insight into habit, embodied orientation, and more durable self-regulation.	Daily practice logs; retreat and program schedules; attendance and adherence records; community rules; and longitudinal practitioner narratives.
Feedback and correction	Practice includes opportunities for correction, supervision, ethical reflection, and adjustment when practice becomes avoidant, self-centered, or destabilizing.	May improve safety and reduce the risk that decentering, compassion, or attention training becomes dissociation, bypassing, or instrumental self-control.	Supervision notes; teacher feedback records; adverse-event protocols; safeguarding procedures; corrective discussions; and community accountability records.

## 3 The ethical-cultural training ecology model

**FIGURE 1:**
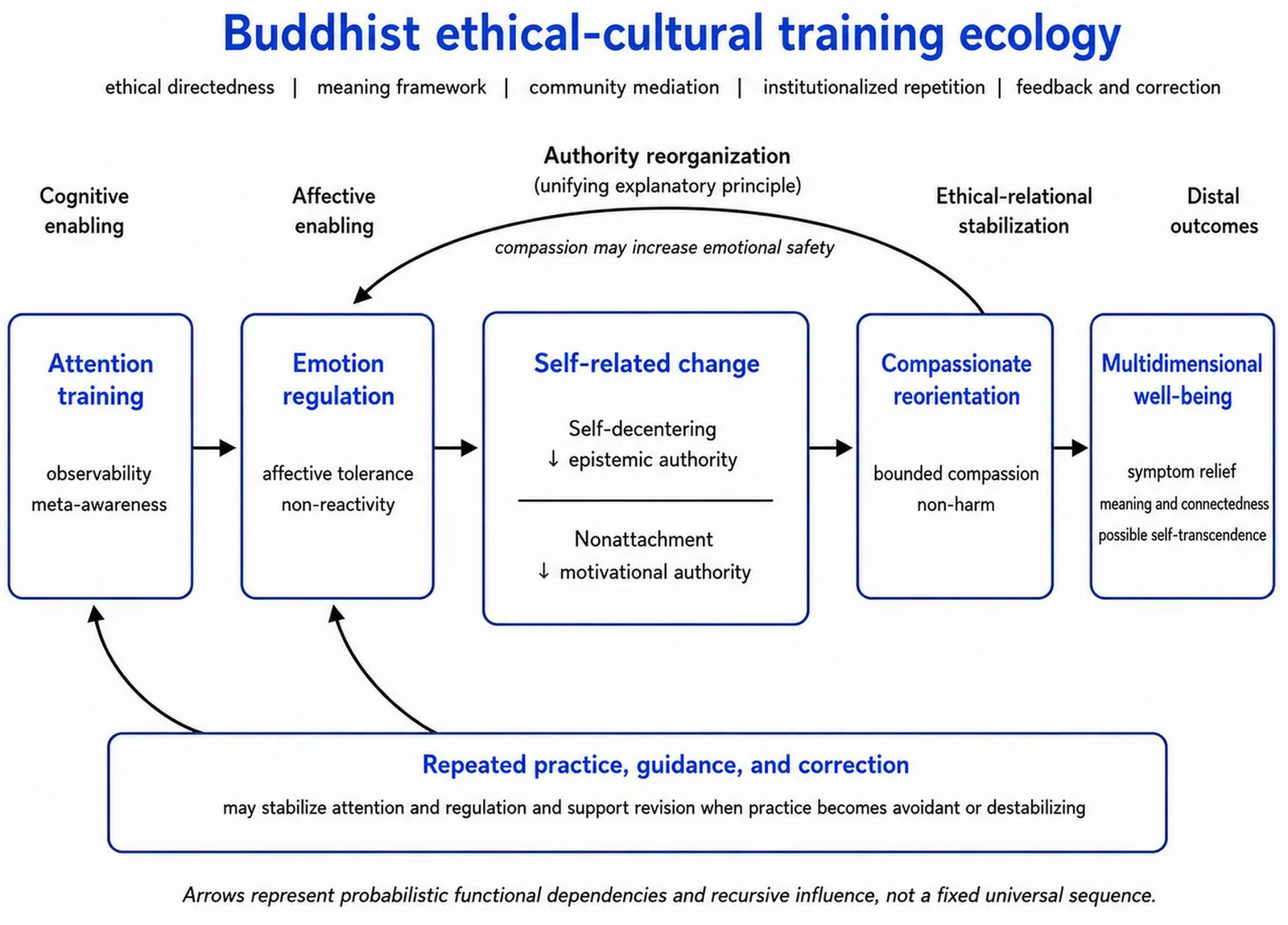
Ethical-cultural training ecology model of Buddhist meditation and psychological well-being. The outer frame represents process-shaping ecological conditions, while the central pathway depicts probabilistic functional dependencies among attention training, emotion regulation, self-decentering and nonattachment, compassionate reorientation, and distal clinical and well-being outcomes. Authority reorganization is depicted as the model’s unifying explanatory principle rather than as an additional mediating mechanism. It summarizes changes in the truth-status and action-guiding force of self-related experience across the functional hierarchy. Compassion and repeated practice, guidance, and correction provide distinct recursive pathways. Self-transcendence is treated as a possible, non-necessary experiential and relational outcome rather than a mediating mechanism. The arrows do not imply a fixed or universally necessary temporal sequence.

Figure 1 organizes the ethical-cultural training ecology model around four analytically distinct but functionally connected components: ecological conditions, a probabilistic functional hierarchy, authority reorganization as the unifying explanatory principle, and distal well-being outcomes. The outer ecology includes ethical directedness, meaning framework, community mediation, institutionalized repetition, and feedback or correction. Within this ecology, attention training, emotion regulation, self-decentering and nonattachment, compassionate reorientation, and distal outcomes are linked through hypothesized functional dependencies. Across this hierarchy, attention supports observability, emotion regulation supports affective tolerability, self-decentering and nonattachment reduce epistemic and motivational authority, and compassionate reorientation provides ethical-relational direction; distal well-being outcomes remain analytically distinct from these process functions. The feedback relation from compassion to emotional safety is distinguished from the stabilizing influence of repeated practice, guidance, and correction; the arrows indicate recursive and probabilistic relations rather than a universal temporal sequence.

### 3.1 The distinctive relational structure of the model

The model’s originality lies in specifying asymmetrical and recursive relations among familiar mechanisms rather than adding another component to mindfulness theory. Its central claim is functional: attention primarily enables observability, regulation increases the tolerability of difficult affect, self-decentering and nonattachment alter different dimensions of self-related authority, and compassion gives the resulting flexibility an ethical-relational direction. These functions are probabilistic rather than mandatory, and they may be strengthened, weakened, or redirected by the surrounding training ecology.

Epistemic authority refers to the degree to which a thought, emotion, bodily sensation, or self-narrative is treated as truthful, self-defining, or reality-disclosing. Motivational authority refers to the degree to which the same experience organizes action through avoidance, grasping, defense, or repetitive rehearsal. The two dimensions can dissociate. A practitioner may no longer regard the thought ‘I am worthless’ as literally true while it continues to drive withdrawal or reassurance-seeking; conversely, its behavioral grip may weaken before its subjective truth value has fully changed. This dissociation is the main incremental claim of the authority framework. Cognitive fusion concerns entanglement with mental content, whereas epistemic authority specifies the truth-status granted to that content; belief conviction indexes endorsement, whereas motivational authority concerns action-guiding force; behavioral reactivity captures responses, whereas motivational authority concerns the experienced content’s capacity to organize those responses. Authority reorganization is therefore proposed as a higher-order explanatory description of changing relations among established mechanisms, not as a separate latent mechanism. Its value depends on whether future studies can demonstrate discriminant and incremental prediction beyond cognitive fusion, belief conviction, experiential avoidance, motivational salience, and behavioral reactivity.

The hierarchy is functional rather than strictly chronological. Later processes may appear early, and compassion may motivate practice from the outset. The model therefore proposes probabilistic dependencies rather than a universal temporal sequence. Attention may increase observability, and emotion regulation may increase affective safety. Self-decentering and nonattachment may alter the truth-status and motivational grip of self-related content. Compassion may then give this loosening an ethical direction while recursively reducing shame and defensive reactivity.

Within this functional hierarchy, each process addresses a distinct condition through which epistemic or motivational authority may be maintained. Attention addresses limited observability by making experience available to awareness. Emotion regulation addresses affective intolerability by making painful experience more bearable for examination. Self-decentering demotes self-narratives from absolute truths to conditioned events, while nonattachment weakens their motivational power to organize grasping, avoidance, defense, or repetitive rehearsal. Compassion may then orient this increased flexibility toward care, non-harm, and ethical response rather than detachment or self-optimization.

The hierarchy also generates failure conditions. If attention is weak, difficult experience may remain too automatic to examine. If affective tolerance is insufficient, decentering may be difficult to sustain or may become destabilizing. If self-decentering and nonattachment remain weak, compassion may remain disconnected from self-related transformation and vulnerable to moral exhortation, sentimental concern, or self-erasing care. If compassion and ethical discrimination are absent, cognitive distance may drift toward cold detachment or dissociation. These are probabilistic vulnerabilities rather than claims that every mechanism is necessary for every practitioner.

### 3.2 The process-shaping role of the ethical-cultural frame

The ethical-cultural frame is treated as a process-shaping condition, not as a universally necessary or inherently superior context. It may influence the purpose of attention, the interpretation of emotional difficulty, and the direction of self-related change. When ethical and wisdom-oriented supports are absent or weak, mindfulness can remain clinically useful, but it may be more readily organized around stress management, productivity, performance optimization, or private self-control (Bodhi, 2011; Van Dam et al., 2018; Berger et al., 2024). Such uses may narrow the scope of practice while leaving grasping, avoidance, self-centeredness, and relational harm comparatively unexamined.

In Buddhist-contextualized practice, non-harm, responsibility, wisdom, and compassion may orient attention toward understanding suffering, weakening reactivity, loosening clinging, and responding with care. Ethical analyses have highlighted the importance of retaining explicit ethical framing in mindfulness implementation (Berger et al., 2024), while experimental work suggests that ethics- and wisdom-based practices may influence prosocial outcomes (Furnell et al., 2025). The model therefore hypothesizes three pathways: the frame may direct what attention is trained to notice; it may shape how fear, anger, shame, and grief are interpreted; and it may orient self-related change toward non-harm, compassion, responsibility, and appropriate boundaries.

These pathways are hypotheses about functional orientation, not claims that Buddhist settings are uniformly safer, more effective, or ethically sufficient. Their effects depend on implementation, accountability, teacher competence, participant vulnerability, and opportunities for corrective feedback. Accordingly, training ecology refers to the configuration through which practices are learned, interpreted, repeated, socially mediated, and corrected, rather than to a set of independent techniques placed side by side.

### 3.3 Operationalizing the training ecology

The five dimensions follow a process-shaping logic rather than an attempt to provide an exhaustive taxonomy. Ethical directedness captures normative orientation, meaning framework captures interpretive organization, and community mediation captures social scaffolding and accountability. Institutionalized repetition captures temporal and procedural stabilization, while feedback and correction capture monitoring, safety, and adaptive adjustment. Together, the dimensions ask what practice is for, how it is interpreted, who supports it, how it is stabilized over time, and how errors or risks are corrected.

For comparative research, the five dimensions should initially be represented as a qualitative, dimension-level profile derived from textual analysis, interviews, observations, and program documents. They are best treated as an initial analytic taxonomy open to refinement through comparative work, not as reflective indicators of a single latent construct. Ordinal ratings may become useful only after explicit anchors, coder training, inter-rater agreement, cross-context interpretability, and decision rules have been established.

These dimensions generate a cautious configurational hypothesis: particular combinations of ethical directedness, meaning framework, community mediation, institutionalized repetition, and feedback and correction may be more likely to support durable self-related and relational transformation than other combinations. No single dimension or simple “more-is-better” continuum is assumed. Safety and accountability should be examined as configuration-level conditions rather than as inherent properties of Buddhist, secular, clinical, educational, or hybrid settings.

The multisite comparative strategy outlined in Section “5 Testable propositions, process markers, and empirical strategies” illustrates how these dimension-level profiles could be examined longitudinally without presuming a single ecological-density continuum.

### 3.4 Attention training as an early enabling practice

Attention training is treated as an early enabling practice because regulation or decentering is less likely when mental events remain unnoticed. Mindfulness of breathing, body scanning, walking meditation, and open monitoring train the ability to sustain attention, detect distraction, and return to the present object of awareness. In cognitive terms, this involves monitoring, conflict detection, executive control, and meta-awareness (Lutz et al., 2008; Tang et al., 2015). In Buddhist terms, attention is not mere concentration; it is the cultivation of wakeful knowing that can discern how experience arises and passes. Greater observability may facilitate emotion regulation by making affective reactions available for naming, toleration, and reinterpretation.

It would be a mistake, however, to make attention do all the theoretical work. In this model, attention is an important enabling condition, but not a sufficient explanation of transformation. A practitioner may become skilled at focusing while remaining defensive, self-fused, or ethically indifferent. Some decontextualized forms of mindfulness may therefore help with stress while leaving deeper patterns of attachment comparatively unchanged; attention opens the field of experience, but insight and compassion may require additional forms of cultivation and ecological support.

### 3.5 Emotion regulation in observational awareness

As attentional observability develops, a second functional problem concerns affect. Meditation may slow the familiar passage from stimulus to appraisal, arousal, narrative elaboration, and reaction. Sensations and feelings need not be treated as orders that must be obeyed; they can be contacted as events that may be named, felt, and watched. This interpretation is compatible with psychological accounts of reappraisal, acceptance, exposure, and response inhibition (Gross, 1998, 2015; Chambers et al., 2009).

Here regulation should not be confused with emotional suppression. Fear, anger, shame, or sadness may remain very much present. What changes is the way they are held: less avoidance, less immediate self-judgment, and less narrative escalation. This helps explain the clinical relevance of mindfulness-based work for depression, anxiety, stress, and relapse vulnerability, where rumination and avoidance often intensify affect rather than resolve it (Keng et al., 2011; Pruessner et al., 2024).

In Buddhist terms, this movement resembles a weakening of craving and aversion. In psychological terms, it would be visible in less rumination, less suppression, lower impulsivity, reduced experiential avoidance, and better tolerance of arousal. It may also facilitate decentering. Strong affect tends to make the self-story feel urgent, absolute, and true; as affect becomes more bearable, the story attached to it may become easier to examine rather than simply believe. Emotion regulation is therefore hypothesized to increase the probability of self-decentering, not to constitute a universal prerequisite.

### 3.6 Self-decentering and nonattachment

Under conditions of greater attentional observability and affective tolerability, self-decentering and nonattachment may alter the status and motivational grip of experience. Self-decentering is a metacognitive shift in which thoughts and feelings are known as conditioned events rather than final descriptions of self or reality (Fresco et al., 2007; Bernstein et al., 2015). Nonattachment is an affective-motivational loosening in which grasping, aversion, and self-identification lose some of their power (Sahdra et al., 2010). The processes overlap in reducing fusion, but they are not interchangeable: decentering changes how experience is known, whereas nonattachment changes how tightly it is held. This distinction is also consistent with work on cognitive defusion, deconstructive self-processing, and self-related mechanisms in mindfulness-based interventions (Hayes et al., 2006; Dahl et al., 2015; Britton et al., 2021). Table 4 summarizes their shared region, distinctive markers, risks, and operational implications.

**TABLE 4:** Distinguishing self-decentering and nonattachment.

Dimension	Self-decentering	Nonattachment
Primary change	Epistemic or metacognitive shift	Affective-motivational loosening
Core question	How is the thought, emotion, or self-narrative known?	How tightly is the thought, emotion, or self-narrative held?
Shared region	Both reduce fusion with mental content and weaken automatic identification.	Both reduce fusion with mental content and weaken automatic identification.
Unique phenomenological marker	The practitioner recognizes: ‘This is a thought or feeling arising.’	The practitioner experiences: ‘This thought or feeling no longer has to pull, command, or define me.’
Risk if isolated	Cognitive distance without affective release; clarity may remain dry or defended.	Emotional numbing, avoidance, or dissociation may be mistaken for release.
Operational implication	Operationally approached through decentering, reperceiving, cognitive defusion, and linguistic markers of self-referential flexibility.	Operationally approached through nonattachment, reduced rumination, reduced experiential avoidance, reduced grasping/aversion, and behavioral flexibility.
Relation to Buddhist concept	A provisional psychological analogue for recognizing mental events as conditioned and non-self-defining.	A provisional psychological analogue for reduced clinging, aversion, and rigid identification; Sahdra et al.’s nonattachment scale provides one operational proxy rather than an exhaustive equivalent.

Meta-analytic evidence supports decentering as a mindfulness-related mechanism associated with psychological problems (Guo, 2024). However, this evidence does not establish that decentering and nonattachment are the same construct, nor that either is sufficient to explain the broader training ecology. Their discriminant validity therefore remains an empirical question. A practitioner may recognize a thought as a thought while remaining emotionally or behaviorally gripped by it; conversely, avoidance, numbing, or dissociation may resemble release without genuine metacognitive awareness. The model predicts stronger and more durable transformation when self-decentering reduces epistemic authority, nonattachment reduces motivational authority, and compassion directs the resulting flexibility toward care rather than cold detachment.

Recent three-level meta-analytic evidence from mindfulness- and acceptance-based interventions for music performance anxiety provides convergent, though indirect, support for the relevance of attentional reallocation and decentering. Zhang et al. (2026) reported significant improvements in anxiety-related outcomes and positive psychological functioning and identified cognitive defusion/decentering, somatic anchoring, acceptance/values clarification, and self-compassion as recurring therapeutic components. Because these mechanism-related conclusions were based on theoretical integration and qualitative synthesis rather than direct mediation tests, they support the plausibility of the present attention-to-decentering pathway without establishing its causal ordering or validating authority reorganization.

### 3.7 Compassionate reorientation

The model would remain ethically under-specified without compassion. Compassionate reorientation is examined here in relation to other-directed compassion, self-compassion, and loving-kindness, while recognizing that these are related but distinguishable constructs. It is not treated merely as an additional positive feeling. In the present model, self-transcendence is treated as a possible experiential and relational outcome of reduced self-fusion and compassionate reorientation, rather than as a mechanism identical to compassion. Compassion matters because Buddhist meditation is concerned with suffering, not only with private distress. As self-fusion weakens, compassion may help prevent decentering from becoming dry detachment and may create more room to respond to suffering without immediately defending the self.

This extension is consistent with several strands of psychological work. Loving-kindness and compassion meditation have been linked to positive emotion, empathy, prosocial motivation, and compassion (Fredrickson et al., 2008; Hofmann et al., 2011; Luberto et al., 2018). Self-compassion research connects kinder self-attitudes with lower self-criticism (Neff, 2003a, b; Gilbert, 2010). Research on self-transcendence and mindfulness-to-meaning adds another layer. Meditation may reshape perceived boundaries of body and self, reduce self-centeredness, and support meaning and well-being (Dambrun and Ricard, 2011; Garland et al., 2015, 2022; Garland and Fredrickson, 2019; Hanley et al., 2020, 2023; Dambrun et al., 2024; De Oliveira et al., 2026).

Within the model, compassion has a double role. It may be strengthened through the coordination of attention, regulation, decentering, and nonattachment because these processes can make suffering easier to recognize without defensive avoidance. It may also motivate practice from the outset and feed back into earlier processes: a more compassionate stance may reduce shame-based reactivity and increase affective safety, allowing difficult material to be approached again with less avoidance or self-attack. This recursive hypothesis distinguishes the present model from linear accounts in which compassion appears only as an outcome.

Bounded compassion is used here as a descriptive relational configuration rather than as a validated unitary latent construct. It would be operationalized through conjoint evidence of compassionate concern, self-compassion, assertiveness or interpersonal effectiveness, and maintained boundaries or reduced self-silencing, rather than through a general compassion score alone.

### 3.8 Multidimensional outcomes and self-transcendence

The outcome domain is multidimensional psychological well-being rather than symptom reduction alone. The model therefore separates several analytic levels of outcome. Proximal capacities include attentional stability and affective tolerance. Intermediate self-related transformations include self-decentering, nonattachment, and reductions in cognitive fusion, rumination, experiential avoidance, and self-criticism. Relational outcomes include bounded compassion, prosocial orientation, and relational flexibility. Distal clinical and well-being outcomes include reduced anxiety, depression, stress, and relapse vulnerability, together with greater meaning, connectedness, equanimity, and possible self-transcendence. Compassion can be analyzed both as a recursive relational process within practice and, when generalized beyond practice, as an interpersonal outcome. Empirical studies should specify the analytic level being measured rather than collapse all changes into a single wellness score (Ryff, 1989; Seligman, 2011; Feng et al., 2025).

Self-transcendence is explicitly distinguished from compassionate reorientation in this framework. It is treated as a possible emergent outcome of reduced self-fusion and expanded relational concern, not as a necessary mediator of well-being. Future studies may operationalize this outcome through measures of perceived body-boundary salience and spatial frames of reference (Hanley et al., 2020), complemented by qualitative reports of diminished self-world separation or broadened relational identification.

## 4 Model application: three practice-anchored theoretical illustrations

This section presents three theory-driven hypothetical illustrations rather than empirical cases. Each illustration is organized around three elements: a predicted pathway derived from the framework, a disconfirming pattern that would weaken the proposed explanation, and a future test through which the pathway could be examined. The illustrations address an intrapersonal problem (anxious self-narrative), an interpersonal problem (resentment), and an ecology-level problem (variation between decontextualized technique and threefold-training practice).

**FIGURE 2:**
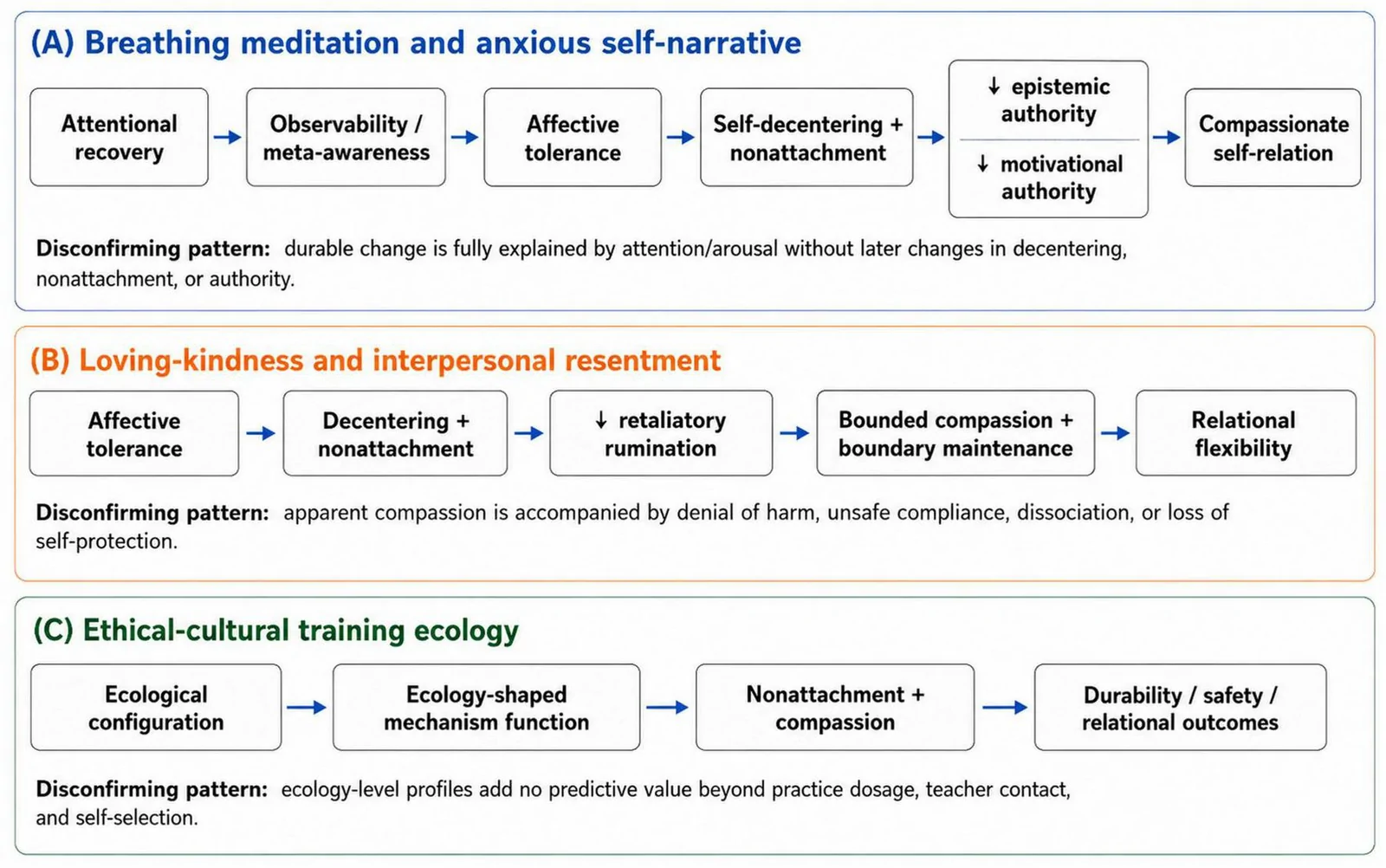
Simplified theory-driven pathways for the three practice-anchored illustrations. Each panel states the predicted pathway and an empirical pattern that would weaken the model. The arrows indicate hypothesized probabilistic relations rather than a fixed temporal sequence.

The illustrations are anchored in recognizable practice logics and selected empirical literatures. Table 5 summarizes the predicted pathways, discriminating functions, and disconfirming patterns in the three theoretical illustrations. The first draws on breath awareness and Anapanasati meditation. The second draws on loving-kindness and compassion practice. The third examines Buddhist-contextualized mindfulness shaped by morality, concentration, wisdom, precepts, community, and feedback (Basu et al., 2025; Hofmann et al., 2011; Ekman et al., 2022; Lee et al., 2017; DeMaranville et al., 2022; Wongpakaran et al., 2022; Zhang and Shi, 2024). These illustrations are deductive stress points for the model, not evidence for it. Figure 2 summarizes the proposed pathways and the empirical patterns that would count against them.

**TABLE 5:** Predicted pathways, discriminating functions, and disconfirming patterns in the three theoretical illustrations.

Illustration	Practice anchor	Predicted pathway	Discriminating model function	Disconfirming pattern / future test
Mindfulness of breathing and anxious self-narrative	Mindfulness of breathing; bodily observation; repeated return from distraction; Anapanasati and satipatthana-style noticing.	Attentional recovery → meta-awareness/observability → affective tolerance → decentering → reduced epistemic authority; nonattachment reduces motivational grip.	Tests whether attentional improvement alone is insufficient for durable change in anxious self-identification.	Weakened if attention/arousal fully explains durable change. Test with EMA, belief/authority ratings, diaries, and longitudinal interviews.
Loving-kindness practice and interpersonal resentment	Metta/karuna cultivation and compassion practice with ethical boundaries.	Affective tolerance → decentering/nonattachment → less retaliatory rumination → bounded compassion + boundary-setting → relational flexibility.	Tests relational transformation beyond private calm and distinguishes compassion from forced forgiveness.	Weakened by denial of harm, unsafe compliance, dissociation, or loss of self-protection. Test with longitudinal compassion, rumination, assertiveness, and boundary indicators.
Buddhist-contextualized mindfulness training ecology	Threefold training of morality, concentration, and wisdom; precepts; teacher/community guidance; repeated practice; correction.	Ecological configuration → altered function of attention/self-related change → nonattachment/compassion → durability, safety, and relational outcomes.	Tests whether ecology predicts downstream function beyond dosage and technique labels.	Weakened if ecology profiles add no value beyond dosage, teacher contact, and self-selection. Test through multisite longitudinal comparison.

### 4.1 Breathing meditation and the anxious self-narrative

The first illustration asks whether the proposed hierarchy can explain anxious self-narrative without reducing breathing practice to relaxation. The practice anchor is mindfulness of breathing, where repeated return to the breath interrupts narrative proliferation and makes experience observable (Analayo, 2003; Bodhi, 2011; Basu et al., 2025).

Predicted pattern. Successful return to the breath should increase observability and meta-awareness, which should make anxious affect more tolerable and increase the probability of self-decentering. Decentering should reduce the epistemic authority of anxious self-narratives, while nonattachment should reduce their motivational grip and create greater scope for compassionate self-relation. Disconfirming pattern. The framework would be weakened if durable improvement in anxious self-identification were fully explained by attentional performance or arousal reduction without subsequent changes in decentering, nonattachment, or authority.

Testable implication. Intensive longitudinal work could assess post-practice attentional recovery and meta-awareness, weekly affective tolerance, and later changes in decentering, belief conviction, avoidance, and action-guiding grip. Time-lagged analyses would be informative if improvements in successful attentional recovery prospectively precede affective tolerance and self-related change rather than merely covary with them. Narrative data could provide complementary markers, such as movement from identity claims (‘I am inadequate’) to process descriptions (‘fear is arising and being observed’).

### 4.2 Loving-kindness practice and interpersonal resentment

The second illustration examines whether the framework can explain relational injury without collapsing compassion into forced forgiveness. The practice anchor is loving-kindness and compassion meditation, which trains goodwill while allowing resentment, hurt, and boundary concerns to remain ethically visible (Hofmann et al., 2011; Luberto et al., 2018; Ekman et al., 2022; Lee et al., 2017).

Predicted pattern. As resentment becomes more observable and affectively tolerable, self-decentering should loosen the injured-self narrative and rigid representation of the other, while nonattachment should reduce retaliatory rumination and the action-guiding force of grievance. Bounded compassion should then predict proportionate action, retained self-protection, and greater relational flexibility rather than indiscriminate forgiveness.

Disconfirming pattern and testable implication. The framework would be challenged if compassion practice systematically predicted denial of harm, unsafe compliance, dissociative detachment, or loss of boundary-setting. Longitudinal and qualitative studies could therefore examine whether increases in compassion are followed by lower retaliatory rumination and greater relational flexibility while assertiveness and self-protection are maintained.

### 4.3 Buddhist-contextualized mindfulness as a threefold-training ecology

The third illustration shifts from individual process to training ecology. Buddhist-contextualized mindfulness is treated as an analytic category referring to mindfulness situated within morality, concentration, and wisdom, together with supports such as precepts, teacher or community guidance, repeated practice, and feedback or correction. The comparison does not oppose East and West or Buddhist and secular settings. It concerns variation in how ethical directedness, meaning framework, community mediation, institutionalized repetition, and feedback or correction are configured across secular, Buddhist-informed, religious, clinical, and hybrid settings.

Predicted pattern. The same nominal attention practice may acquire different downstream functions when embedded in different ecological configurations. Configurations with stronger ethical directedness and corrective feedback may be more likely to connect attention with non-harm, accountability, nonattachment, compassion, and practice safety, whereas configurations organized primarily around stress management may produce substantial attentional or symptomatic benefits without the same relational trajectory. This is a probabilistic contrast, not a claim that Buddhist settings are inherently superior. Five Precepts research, engaged Buddhist mindfulness, and ethics- and wisdom-based studies provide practice anchors for treating these dimensions as psychologically relevant (DeMaranville et al., 2022; Wongpakaran et al., 2022; Zhang and Shi, 2024; Furnell et al., 2025). No configuration is assumed to be uniformly optimal; its safety and effectiveness may depend on fit with participant vulnerability, clinical needs, practice goals, and available support.

Disconfirming pattern and testable implication. The ecology claim would be weakened if dimension-level profiles failed to predict practice meaning, safety, durability, or relational outcomes after controlling practice intensity, teacher contact, participant vulnerability, baseline religiosity, cultural background, and self-selection, or if a simpler dosage model performed equally well. Multisite longitudinal comparison is therefore more informative than a predetermined Buddhist-versus-secular contrast.

### 4.4 Comparative reading of the three illustrations

Taken together, the three illustrations test the framework at intrapersonal, interpersonal, and ecological levels. Their purpose is not to confirm the model but to expose it to alternative explanations. Support would require the predicted temporal or incremental relations to appear beyond simpler attentional, regulatory, or dosage accounts; repeated failure of these discriminating predictions would require the framework to be narrowed or revised.

## 5 Testable propositions, process markers, and empirical strategies

This section translates the model into five testable propositions, observable process markers, and implementable empirical strategies. Each proposition is framed as an expected prospective, mediational, incremental, or configuration-level relation rather than as a finding of the present article. The purpose is to distinguish the model from additive, mindfulness-to-meaning, and clinical-skills alternatives. Table 6 lists process markers and candidate operational indicators for future empirical inquiry.

**TABLE 6:** Process markers and candidate operational indicators for future empirical inquiry.

Construct	Process marker	Candidate operational indicator	Possible material
Attention training	Successful detection of distraction and recovery to the practice object while distinguishing awareness from thought flow.	Return-to-object latency or success rate; sustained-attention indices; meta-awareness ratings; practice logs.	EMA/post-practice reports; practice apps; laboratory tasks; teacher observation.
Emotion regulation	Naming and tolerating fear, anger, shame, or grief without immediate avoidance, suppression, or impulsive reaction.	Affect-tolerance measures; emotion-regulation difficulties; EMA reactivity; physiological arousal.	EMA; clinical notes; body-focused interviews; psychophysiological recordings.
Self-decentering	Shift from identity claims to process descriptions and reduced treatment of self-related content as literal truth.	Decentering/reperceiving/defusion measures; belief-conviction ratings; linguistic self-reference markers.	Narrative interviews; session transcripts; reflective diaries; language-based coding.
Nonattachment	Reduced grasping, aversion, compulsive rehearsal, and identity-based defense without emotional numbing.	Nonattachment; rumination; experiential avoidance; behavioral flexibility; approach-avoidance indicators.	Practice narratives; conflict diaries; behavioral tasks; longitudinal self-report.
Compassion and bounded compassion	Self-kindness, reduced retaliation, recognition of shared vulnerability, and boundary-setting without self-erasure.	Conjoint profile: Self-Compassion Scale; Compassion Scale; prosocial behavior; assertiveness/interpersonal effectiveness; boundary maintenance; reduced self-silencing. Not a general compassion score alone.	Therapist reports; interpersonal narratives; practitioner diaries; observation.
Training ecology	Configuration of ethical directedness, meaning framework, community mediation, institutionalized repetition, and feedback/correction.	Dimension-level profiles from Table 3; practice frequency; teacher contact; community participation; precept/ethical framing.	Program documents; teacher interviews; community observation; practice records.
Authority reorganization	Dissociation or coordinated change between the truth-status and action-guiding force of self-related experience.	Epistemic: belief conviction, cognitive fusion/defusion, truth-status ratings. Motivational: action-guiding grip, motivational salience, avoidance/approach behavior, behavioral reactivity.	EMA with thought/conviction/action ratings; narrative coding; behavioral approach-avoidance tasks; longitudinal self-report.

For clarity, each proposition specifies a focal predictor or configuration, an outcome, relevant covariates or competing constructs, and a pattern that would weaken the claim. The statistical forms below are illustrative rather than prescriptive; they make the theoretical commitments explicit without implying that one design is uniquely appropriate.

Because the dependencies are probabilistic, support requires more than contemporaneous correlation. Later processes should be prospectively more likely when earlier capacities or ecological supports are present, and the model should add explanatory value beyond practice dosage, expectancy, baseline mindfulness, symptoms, and established constructs such as cognitive fusion or psychological flexibility.

**Proposition 1:** In intensive longitudinal designs, within-person increases in successful attentional recovery will prospectively predict greater affective tolerance at subsequent assessments after adjustment for baseline mindfulness, baseline symptoms, expectancy, and practice dosage. Increased meta-awareness is expected to account for part of this prospective association. The proposition would be weakened if the time-lagged effect were absent or fully accounted for by dosage, nonspecific treatment factors, or baseline differences.

**Proposition 2:** Affective tolerance will partially mediate the prospective association between earlier attentional stabilization and later self-decentering. A lagged indirect effect is expected even after adjustment for prior decentering and relevant symptom change, without implying that affective tolerance is universally necessary. The proposition would require revision if the reverse pathway were consistently stronger, if decentering reliably preceded affective tolerance, or if both were better explained by an alternative mechanism.

**Proposition 3:** Self-decentering and nonattachment will be empirically distinguishable and will show different incremental associations. After controlling for decentering and cognitive fusion, nonattachment will predict lower grasping, rumination, experiential avoidance, identity-based defense, and greater behavioral flexibility. In parallel, reductions in epistemic authority should be more closely associated with changes in metacognitive stance and belief conviction, whereas reductions in motivational authority should be more closely associated with nonattachment and reduced action-guiding grip. The proposition would be challenged if these constructs showed no distinguishable psychometric, linguistic, behavioral, phenomenological, or predictive markers.

**Proposition 4:** Compassion will function both as a relational outcome and as a recursive stabilizer. After controlling prior emotion regulation, self-decentering, and nonattachment, increases in compassion should predict subsequent emotional safety, lower shame-based reactivity, continued engagement with difficult experience, and relational flexibility while boundary-setting is maintained. The proposition would be weakened if compassion added no prospective prediction or if apparent compassion were better explained by avoidance, compliance, self-silencing, or self-erasure.

**Proposition 5:** Dimension-level ecological configurations will predict 6-month compassionate engagement, relational flexibility, relational durability, and practice-safety trajectories beyond practice intensity, teacher contact, baseline outcomes, and self-selection. A preregistered contrast could compare programs matched on practice dosage but differing in ethical directedness and consistent corrective feedback. Among programs matched on practice dosage, configurations characterized by stronger ethical directedness and consistent corrective feedback are expected to show greater 6-month compassionate engagement and relational flexibility, while allowing these associations to vary across cultural and motivational contexts. Directly measured self-construal, religious orientation, and practice motivation may also be examined as moderators of configuration-outcome associations. The proposition would be weakened if ecology profiles add no incremental predictive value beyond dosage and baseline covariates or if configuration differences fail to relate to durability, safety, or relational outcomes.

A recent three-level meta-analysis also found that intervention dose did not significantly moderate the main effects of mindfulness- and acceptance-based interventions in the specific context of music performance anxiety (Zhang et al., 2026). This context-specific null result does not imply that practice quantity is irrelevant; rather, it reinforces the value of testing whether process configuration and intervention quality explain variance beyond simple dosage. For the present model, the relevant empirical question is whether dimension-level ecology profiles contribute incremental prediction after practice intensity and other selection factors are controlled.

The propositions can be tested through complementary longitudinal, experience-sampling, narrative, psychometric, behavioral, and multisite designs. The following examples illustrate how the statistical claims could be implemented while preserving the framework’s probabilistic character.

Propositions 1 and 2 could be examined in an 8-week program with post-practice EMA and weekly assessments. Post-practice reports could record successful attentional recovery, mind-wandering, meta-awareness, practice duration, and immediate affective reactivity; weekly measures could assess affective tolerance and decentering. Multilevel time-lagged mediation could test whether attentional recovery predicts later affective tolerance and whether meta-awareness and affective tolerance account for subsequent self-decentering, with baseline symptoms, expectancy, prior meditation experience, and dosage entered as covariates.

Propositions 3 and 4 could be tested with at least three longitudinal waves combining psychometric and narrative evidence. Confirmatory or latent-variable models could evaluate whether decentering, nonattachment, and the proposed authority dimensions are empirically distinguishable; hierarchical or structural models could then test incremental prediction beyond cognitive fusion, belief conviction, experiential avoidance, and prior emotion regulation. Parallel diary or interview coding could distinguish recognizing mental content as an event from reduced compulsion to defend, avoid, or rehearse it. Cross-lagged analyses, where design and sample size permit, could test whether compassion predicts later emotional safety rather than merely appearing as a terminal outcome.

Proposition 5 requires a multisite longitudinal design rather than a predetermined ‘low-density secular’ versus ‘high-density Buddhist’ contrast. Participants (Level 1) would be nested within programs or sites (Level 2), and sites would be profiled separately on ethical directedness, meaning framework, community mediation, institutionalized repetition, and feedback or correction rather than collapsed into a single additive ecology score. Multilevel models could test whether prespecified dimension-level profiles and preregistered configuration contrasts predict 6-month compassionate engagement, relational flexibility, durability, and adverse-event patterns after adjustment for baseline outcomes, practice intensity, teacher contact, religiosity, self-construal, participant vulnerability, and self-selection. Incremental configuration-level prediction beyond a dosage-only comparator would support the ecological claim; null or nonincremental configuration effects would weaken it. Matched-sample or propensity-based sensitivity analyses could further probe selection bias.

## 6 Discussion

### 6.1 Theoretical implications

As summarized in Figure 1, the model conceptualizes transformation as a reorganization of the epistemic and motivational authority of experience rather than as an accumulation of isolated skills. The authority formulation is intended to add explanatory precision only if the two dimensions can diverge and contribute information beyond established constructs. A self-critical narrative, for example, may lose truth-status before it loses behavioral grip, or may lose action-guiding force before its subjective credibility fully changes. This distinction links the functional hierarchy to observable dissociations and prevents authority reorganization from functioning as a purely retrospective label. The broader theoretical contribution remains relational: observability, affective tolerability, loosened self-identification, nonattachment, and ethically directed compassion are hypothesized to have asymmetrical roles whose operation may vary across training ecologies.

Culturally, the framework treats ethical-cultural conditions as variable configurations rather than as fixed properties of Buddhist or secular settings. This approach permits comparison of how ecological supports are retained, translated, weakened, or absent without presuming a simple Buddhist-versus-secular opposition; its cross-context implications are considered further in Section “6.3 Implications for positive and cross-cultural psychology”.

Methodologically, the model links selected Buddhist concepts to provisional qualitative and mixed-method indicators. Mindfulness, concentration, insight, nonattachment, compassion, and ethical orientation are connected to narrative markers, linguistic shifts, practice routines, interpersonal changes, and ecology-level features without being reduced to equivalent psychological constructs. This approach allows future studies to examine Buddhist meditation without collapsing it into a single scale score or treating Buddhist context as merely decorative background.

The model does not require every mechanism or all five ecological dimensions to be present in every instance. Its minimal empirical core is relational rather than component-based: at least one enabling process should increase the observability or tolerability of experience, at least one self-related process should reduce its epistemic or motivational authority, and ecological conditions should be capable of modifying the function, durability, or direction of these changes. The absence of compassion or particular ecological supports would therefore predict a narrower or differently configured outcome profile rather than automatically invalidate the model.

### 6.2 Clinical implications

For clinical psychology, the model suggests that mindfulness-based interventions should be evaluated beyond stress reduction or symptom relief. Programs may need to consider whether attention training is accompanied by affective tolerance, self-decentering, nonattachment, self-compassion, and compassion for others, because these processes may shape whether distress is merely noticed or whether identification with distress is transformed.

The second illustration translates this point into the idea of bounded compassion. Clinically, compassion should not mean tolerating repeated harm, silencing moral injury, or collapsing one’s needs into the needs of others. Bounded compassion is better understood as a disciplined relational capacity that integrates care, clarity, non-harm, boundary-setting, and self-protection. This formulation is compatible with assertiveness, interpersonal effectiveness, Compassion Focused Therapy, and evidence on compassion-based interventions (Gilbert, 2010; Linehan, 2015; Kirby et al., 2017; Speed et al., 2018).

The clinical caution is that attention, decentering, and compassion-oriented practices are not automatically safe. Attention practice can surface difficult material before adequate emotional support is available; decentering may be confused with dissociation or cold detachment; and poorly scaffolded compassion-oriented practice may evoke empathic distress, overextension, or self-erasing care when affective support and boundaries are weak. Integrated programs may therefore require individualized pacing, informed consent, appropriate screening, and ongoing monitoring, particularly for trauma-exposed or clinically vulnerable participants (Farias et al., 2020; Matko and Van Dam, 2026).

### 6.3 Implications for positive and cross-cultural psychology

From the perspective of positive psychology, the model shifts meditation research from symptom reduction toward flourishing, meaning, relational openness, and ethically bounded compassion. It distinguishes proximal capacities such as attentional clarity and affective tolerance, intermediate transformations such as reduced rumination and nonattachment, relational outcomes such as bounded compassion and relational flexibility, and distal outcomes such as meaning, equanimity, and possible self-transcendence. These levels should be examined separately rather than collapsed into a general wellness score.

From the perspective of cross-cultural psychology, the model treats ecological configuration as a comparative profile rather than as a Buddhist-versus-secular binary. Researchers can compare how ethical directedness, meaning framework, community mediation, institutionalized repetition, and feedback or correction are retained, translated, weakened, or transformed across Buddhist, secular, clinical, educational, and hybrid settings. This comparison does not imply that Buddhist settings are inherently safer or superior: highly structured settings may still involve authoritarianism, group conformity, spiritual bypassing, or inadequate responses to vulnerability when accountability and corrective feedback are absent.

Within this comparative framing, the selected Chinese-language sources are supplementary interpretive resources rather than substitutes for the international mindfulness literature or representatives of East Asian Buddhism as a whole. They identify culturally situated assumptions about selfhood, suffering, cultivation, and nonattachment while leaving empirical and cross-context validity for future research. Drawing on work on independent and interdependent self-construals (Markus and Kitayama, 1991), future studies should test whether directly measured self-construal alters the magnitude or direction of associations among ecology profiles, self-related change, and relational outcomes. Religious orientation and practice motivation may be examined in the same way. These variables should be measured directly rather than inferred from nationality, ethnicity, or religious affiliation.

## 7 Limitations and boundary conditions

The first limitation is historical and doctrinal. Buddhist meditation is not one uniform practice. Theravada lineages; diverse Mahayana traditions, including Chan/Zen and Pure Land; Vajrayana traditions; and contemporary secular adaptations differ in goals, techniques, doctrines, and social settings. The model identifies selected psychological mechanisms, but it should not be read as a claim that all Buddhist practices work in the same way or that all traditions give equal weight to mindfulness of breathing, nonattachment, loving-kindness, compassion, and the threefold training.

The model is most applicable to Buddhist-derived practices in which attentional cultivation, affective regulation, insight into self-related processes, nonattachment, and compassion are explicitly or implicitly coordinated. It is less directly applicable to practices whose primary structure is devotional, ritual, visualization-based, esoteric, or sudden-awakening oriented unless those practices can be shown to involve comparable functional relations. This boundary is important because a cross-traditional psychological abstraction should not be treated as a complete doctrinal map of Buddhism. Future comparative work could examine whether these functional dependencies appear in selected Theravada-derived insight practices and Chan/Zen contemplative settings while avoiding the assumption that doctrinally different practices instantiate identical psychological processes.

Second, the model is theoretical, and both its proposed mechanism relations and its practice-anchored illustrations require qualitative and empirical validation. The illustrations establish interpretive plausibility rather than providing evidence from participant datasets, formal measurements, clinical records, or secondary analyses. Future research should examine whether practitioner narratives, clinical formulations, meditation diaries, and culturally situated practice accounts display the proposed probabilistic dependencies or reveal alternative pathways.

Third, the conceptual correspondences between Buddhist and psychological categories remain partial. Non-self is not identical to decentering; compassion in Buddhist ethics is not identical to empathy; and liberation is not identical to subjective well-being. These correspondences should therefore be treated as provisional analytic analogues rather than direct translations or empirical equivalences. Their use requires conceptual transparency and methodological restraint.

A related and more fundamental limitation concerns the level of abstraction itself. Translating culturally embedded Buddhist practices into psychological mechanisms may privilege categories developed within contemporary psychological science and obscure ethical, soteriological, relational, or lineage-specific meanings. Treating the proposed correspondences as provisional analogues reduces, but does not eliminate, this risk.

Fourth, the status of epistemic and motivational authority remains theoretical. They are proposed as higher-order organizing dimensions rather than validated latent constructs or established measurement domains. Future work should first test whether their proposed dissociation can be detected across self-report, narrative, and behavioral indicators, and then whether they provide incremental prediction beyond cognitive fusion, belief conviction, appraisal, motivational salience, experiential avoidance, and behavioral reactivity. If established constructs fully explain the same patterns, authority reorganization should be treated as a descriptive shorthand rather than a distinct explanatory contribution and the model would require revision.

Fifth, meditation can produce adverse effects in some contexts, including anxiety, dissociation, traumatic memory activation, or destabilization during intensive practice. Research on meditation-related adverse events indicates that adverse-event monitoring, screening, informed consent, and attention to individual vulnerability are necessary components of responsible implementation (Farias et al., 2020; Matko and Van Dam, 2026). Future research should examine moderators such as trauma history, dissociation, practice intensity, teacher competence, social support, and cultural fit.

Sixth, the proposed mechanism relations may be culturally moderated. Future studies should test directly measured self-construal, practice motivation, religious orientation, and related contextual variables as moderators of mechanism and ecology-outcome associations. Nationality, ethnicity, cultural affiliation, or religious identity should not be used as psychological proxies for these constructs.

Finally, the five ecology dimensions constitute an initial analytic taxonomy rather than a validated scale, additive score, or single continuum of ecological ‘density.’ Their organizing logic is functional: normative orientation, interpretive organization, social scaffolding, temporal-institutional stabilization, and corrective regulation. Their distinctiveness, overlap, and interactions remain to be established. The taxonomy should be revised if comparative data identify redundant dimensions, additional process-shaping conditions, or configurations that better predict meaning, safety, durability, and relational outcomes.

## 8 Conclusion

This article advances the ethical-cultural training ecology model as a hypothesis-generating account of Buddhist meditation and psychological well-being. Its contribution is not a new inventory of mechanisms but a relational proposal: established mechanisms may become functionally differentiated through asymmetrical dependencies and may operate differently across ethical-cultural configurations. Attention supports observability, regulation supports affective tolerability, self-decentering and nonattachment target different dimensions of self-related authority, and compassion provides ethical-relational direction while potentially stabilizing earlier processes.

The model is intentionally falsifiable. It should be narrowed or revised if authority reorganization adds no explanatory value beyond established constructs or if self-decentering and nonattachment cannot be meaningfully distinguished. Revision would also be required if compassion fails to predict later emotional safety while preserving appropriate boundaries, or if ecology profiles do not explain durability, safety, or relational outcomes beyond simpler dosage and skills accounts. The five propositions and their operational strategies provide a route for longitudinal, multimethod, and cross-context evaluation rather than evidence that these claims are already established.

## Data Availability

The original contributions presented in the study are included in the article/supplementary material, further inquiries can be directed to the corresponding author.
